# A Differential Pressure Sensor Coupled with Conductance Sensors to Evaluate Pressure Drop Prediction Models of Gas-Water Two-Phase Flow in a Vertical Small Pipe

**DOI:** 10.3390/s19122723

**Published:** 2019-06-17

**Authors:** Yuan-Rong Deng, Ning-De Jin, Qiu-Yi Yang, Da-Yang Wang

**Affiliations:** School of Electrical and Information Engineering, Tianjin University, Tianjin 300072, China; dengyr@tju.edu.cn (Y.-R.D.); qyyang@tju.edu.cn (Q.-Y.Y.); wangdayang@tju.edu.cn (D.-Y.W.)

**Keywords:** gas-water two-phase flow, pressure drop prediction models, differential pressure coupled with conductance sensor, two-phase flow mixture friction factor

## Abstract

In the process of production logging to evaluate fluid flow inside pipe, logging tools that force all flow to pass through a small measuring pipe are commonly utilized for measuring mixture density. For these logging tools, studying the fluid flow phenomenon inside the small diameter pipe and improving the prediction accuracy of pressure drop are beneficial to accurately measure mixture density. In this paper, a pressure drop prediction system is designed based on a combination of an eight-electrode rotating electric field conductance sensor (REFCS), plug-in cross-correlation conductance sensor, and differential pressure sensor. This combination overcomes the limitation of the existing pressure drop prediction model that the inlet flow velocity needs to be known. An experiment is conducted in a flow loop facility with 20 mm inner diameter small pipe. The responses of the combination sensors are collected. The REFCS is used to identify flow pattern and measure water holdup. During which five flow patterns are identified by recurrence plot method, i.e., slug flow, bubble flow, churn flow, bubble-slug transitional flow, and slug-churn transitional flow. The mixture velocity of two-phase flow is determined by the plug-in conductance sensor. The differential pressure sensor provides a differential pressure fluctuation signal. Five models of prediction of pressure drop are evaluated. The mixture friction factor of gas-water two-phase flow is obtained by a fitting method based on the measured parameters and flow pattern identification using the optimal model. Then, the pressure drop can be predicted according to the measurement results of a conductance sensor and fitting relationship. The results of pressure drop prediction show that the model proposed by Ansari et al. presents a higher accuracy compared with the other four differential pressure models with the absolute average percentage deviation (AAPD) of less than 2.632%. Moreover, the accuracy of pressure drop prediction of the Zhang et al. model is improved by using the mixture friction factor.

## 1. Introduction

Production logging traditionally encompasses a number of well logging techniques running on the completed production wells. The purpose of production logging is to evaluate fluid flow inside the pipe. The best devices currently available to measure fluid density in low flowrate gas-water flows are tools that force all flow to pass through a small measuring chamber (pipe) containing a fluid densitometer. When the flow is forced into a much smaller diameter flow path inside the tool, the fluid velocities are increased enough to make the flow steam relatively homogeneous. However, it is still a problem to investigate the flow characteristics of mixture fluid in small diameter pipe. Besides, accurate prediction of pressure drop under small diameter pipe is beneficial to the effective measurement of fluid density. Moreover, the flow velocity and flow pattern need to be known in most pressure drop prediction, which are commonly difficult to measure in downhole environment. Therefore, it is still a great challenge to design a combination sensor system for accurately predicting pressure drop of gas-water two-phase flow in a vertical small pipe.

Differential pressure sensor coupled with conductance sensor can better explore the flow characteristics of two-phase flow because of the abundant measurement parameters. The conductance sensor is sensitive to the changes in fluid field and has high measurement accuracy. Moreover, multiple-electrode conductance sensors such as four-sector conductance sensors [1,2], and rotating electrical field conductance sensors [3,4,5] showed great advantages in flow parameter measurement, as they obtained flow information from multiple channels. The plug-in conductance sensor is a cross-correlation flowmeter with two ring-shape conductance sensors embedded on the center body based on cross-correlation method [6,7,8,9], which is valid for mixture velocity measurement with high measurement accuracy. The combination of various sensors can provide more measurement parameters. Jin et al. [10] combined Electrical Resistance Tomography (ERT) and differential pressure sensors to solve the problem that the application of ERT is limited to the measurement of phase hold-ups in a three-phase system. Areeba et al. [11] used Electrical Capacitance Tomography (ECT) and differential pressure technique on a flow loop to overcome the disruption of industrial processes. Jia et al. [12] combined the differential pressure sensor with ERT and Wire-Mesh Sensor (WMS) to achieve accurate measurement of gas-water two-phase vertical flow void fraction.

Differential pressure sensors have been widely applied to the measurement of gas-water two-phase flow. Prediction models of pressure drop have been formulated in detail by many researchers. Early studies about pressure drop prediction directly calculated mixture density with ignoring slippage effect in upward two-phase flow [13,14,15,16]. Poettmann et al. [17] founded the relationship between pressure drop energy loss and friction factor under upward two-phase flow with low flow rate. Baxendell et al. [18] modified the energy loss curves with high flow rate. While, treating two-phase vertical flow as a homogeneous single-phase flow often results in poor accuracy of pressure drop prediction. In order to solve this problem, Hagedorn et al. [19] established an empirical method for pressure drop prediction based on liquid holdup and friction factor. However, empirical formula still cannot guarantee the accuracy of pressure drop prediction under various flow patterns. Empirical correlations for predicting pressure drop based on identifying bubble flow, slug flow, annular flow, and transition flow were established [20,21]. Asheim [22] defined water holdup and wall friction by three independent parameters for different flow patterns. The mechanism model is more adaptable to predict pressure drop in upward gas-water two-phase flow because of its clearly physical meaning [23,24,25]. Hasan-Kabir [26] predicted pressure drop through the relationship between homogeneous Reynolds number and friction factor based on flow pattern identification and gas holdup prediction. Ansari et al. [27] determined the relationship between Reynolds number and friction factor according to two-phase vertical flow mixture density. Zhang et al. [28,29] predicted slug liquid holdup by pressure drop, which is based on a balance between the turbulent kinetic energy of the liquid phase and the surface free energy of dispersed spherical gas bubble. Moreover, the two-fluid model [30,31] was put forward based on the conservation of mass and momentum of gas-liquid two-phase.

In this study, a pressure drop prediction system based on a combination of differential pressure sensor and conductance sensor is proposed to predict the pressure drop in upward gas-water two-phase flow. For logging tools that force all flow to pass through a small measuring pipe to measure mixture density, studying the fluid flow phenomenon inside the small diameter pipe and improving the prediction accuracy of pressure drop are significant. An experiment is conducted in a flow loop facility with a 20 mm inner diameter small pipe. The response of REFCS is used to identify flow pattern and measure water holdup. Meanwhile, the mixture velocity of two-phase flow is determined by the plug-in conductance sensor. Then the mixture friction factor of two-phase flow is obtained. The results show that the model proposed by Ansari et al. presents a higher pressure drop prediction accuracy compared with other four differential pressure model. Moreover, the accuracy of pressure drop prediction of the Zhang et al. model is improved by using the mixture friction factor. In conclusion, a differential pressure sensor coupled with a conductance sensor for predicting pressure drop has advantages in improving the accuracy of pressure drop.

## 2. Measurement System and Experiment Setup

### 2.1. Measurement System

**Figure 1:**
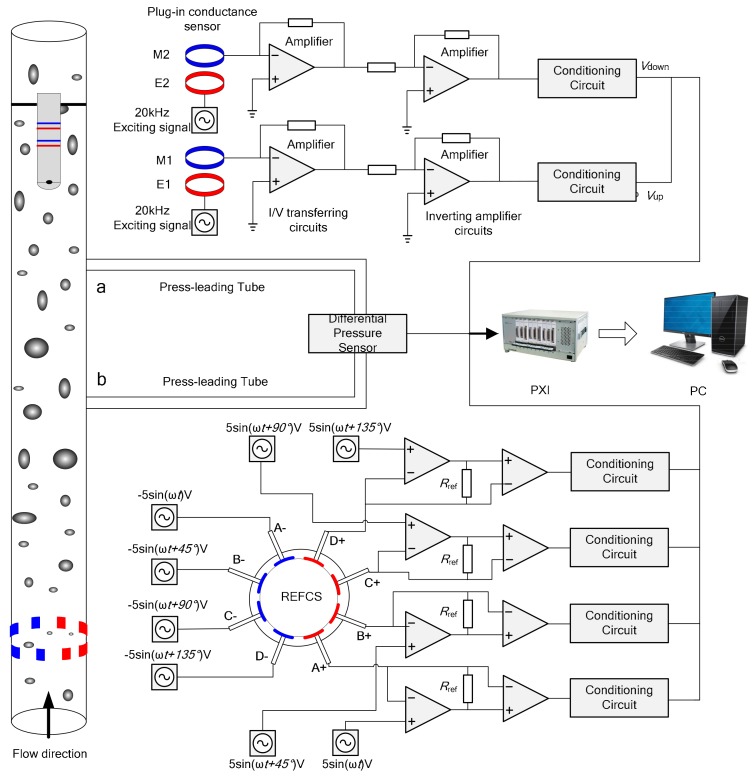
Measurement system of plug-in conductance sensor, differential pressure sensor and rotating electric field conductance sensor (REFCS).

The measurement system of the combination sensors consisting of plug-in conductance sensor, differential pressure sensor and REFCS is shown in Figure 1. When mixture fluid flows in vertical pipe, the differential pressure between a and b pressure ports is transferred into differential pressure sensor by press-leading tubes filled with water. The differential pressure sensor converts pressure signals into voltage signals and sampled by data acquisition system. On the basis of Bernouli equation, the differential pressure Δ*P_ab_* between a and b can be described as follows:

(1) $$\Delta P_{ab}=\Delta P_{e}+\Delta P_{f}+\Delta P_{acc}+\Delta P_{j},$$

where Δ*P_e_*, Δ*P_f_*, Δ*P_acc_*, and Δ*P_j_* are total elevation pressure drop, frictional pressure drop, acceleration pressure drop and local pressure drop, respectively. Considering the short height between two pressure ports, Δ*P_acc_* and Δ*P_j_* can be neglected.

Therefore, the total elevation pressure drop Δ*P_e_* is given as:

(2) $$\Delta P_{e}=\Delta P_{ab}-\Delta P_{f}=(\rho_{y}-\rho_{m})gh-\Delta P_{f},$$

where *ρ_y_*, *ρ_m_*, *g* as well as *h* are the density of press-leading liquid(water), the density of mixing fluid, the acceleration of gravity, and the height between *a* and *b*, respectively.

The configurations and exciting methods of REFCS with eight electrodes [32] and the measurement system of eight electrodes conductance sensor are shown in Figure 1. The sensor consists of four pairs of electrodes uniformly flush-mounted on the same cross section of pipe inner wall, so there is no disturbance to the fluid. Analog sinusoidal signals with 10 V peak-to-peak and 20 kHz different initial phases are applied to the electrodes. Each pair of electrodes is placed opposite to each other, while the phase difference of exciting signals between adjacent electrodes is 45°. Then a rotating electric field is generated to sweep across the test section of pipe. When mixture fluid flows through the detection field, fluctuate signals of the sensor is acquired, and the water holdup could be calculated by normalizing conductance signals.

The relationship between the conductivity of gas-water two-phase flow and the holdup of dispersed phase in mixture fluid can be expressed by the Maxwell [33]:

(3) $$\frac{\sigma_{m}}{\sigma_{w}}=\frac{2(1-Y_{s})}{2+Y_{s}},$$

where *Y_s_*, *σ_m_* and *σ_w_* are the dispersed phase holdup, the conductivity of two-phase mixture and the conductivity of water, respectively. As water holdup *Y_w_* is equal to 1 − *Y_s_*, and the response of REFCS is proportional to the conductivity of mixture fluid, *Y_w_* can be formulated as:

(4) $$Y_{w}^{}=\frac{3}{1+2V_{w}/V_{m}},$$

where *V_m_* and *V_w_* are the sensor response to *σ_m_* and *σ_w_*.

Cross-correlation method is applied to realize velocity measurement by using the similarity of random signals generated by the internal flow of fluid. The structure of plug-in cross-correlation conductance sensor which is used to measure mixture velocity of fluid and the measurement system of plug-in conductance sensor is shown in Figure 1. The distance between upstream sensor and downstream sensor *L* is 30 mm. When the fluid flows through the upstream and downstream sensors, the variation of mixture conductivity changes the conductance between exciting electrode and measuring electrode of each sensor. By detecting the variation of conductance, the upstream signal and downstream signal are obtained which are used for calculating cross-correlation velocity. Analog sinusoidal signals with 4 V peak to peak value, 20 kHz frequency are applied to the exciting electrodes E1 and E2, and the currents in the measuring electrodes M1 and M2 are transformed into voltage signals using I/V transferring circuits and inverting amplifier circuits. The upstream signal is *x(t)*, the downstream signal is *y(t)*. Suppose the structure of mixture fluid flowing through the upstream sensor and downstream sensor has no change. Therefore, *x(t)* and *y(t)* are cross-correlation signals which satisfies the relationship of the following formula:

(5) $$x\left(t\right)=y\left(t+\tau_{0}\right),$$

where *τ*_0_ is the delay time for fluid flowing from the upstream sensor to the downstream sensor, also named transit time. According to cross-correlation theory, the correlation function expressions of *x(t)* and *y(t)* are as follows:

(6) $$R_{xy}\left(\tau \right)=\underset{T\to \infty }{\lim}\frac{1}{T}\int_{0}^{T}x\left(t\right)y\left(t+\tau \right)dt,$$

where *R_xy_*(*τ*) is a cross-correlation function, indicating the relationship and correlation of *x(t)* and *y(t)*.

When *R_xy_(τ)* takes its maximum, the value of time delay *τ* is the transit time *τ*_0_. According to the transit time and the distance *L* between upstream sensor and the downstream sensor, the cross-correlation velocity *U_cc_* of mixture fluid could be calculated as follows:

(7) $$U_{cc}=L/\tau_{0},$$

### 2.2. Experimental Facility

**Figure 2:**
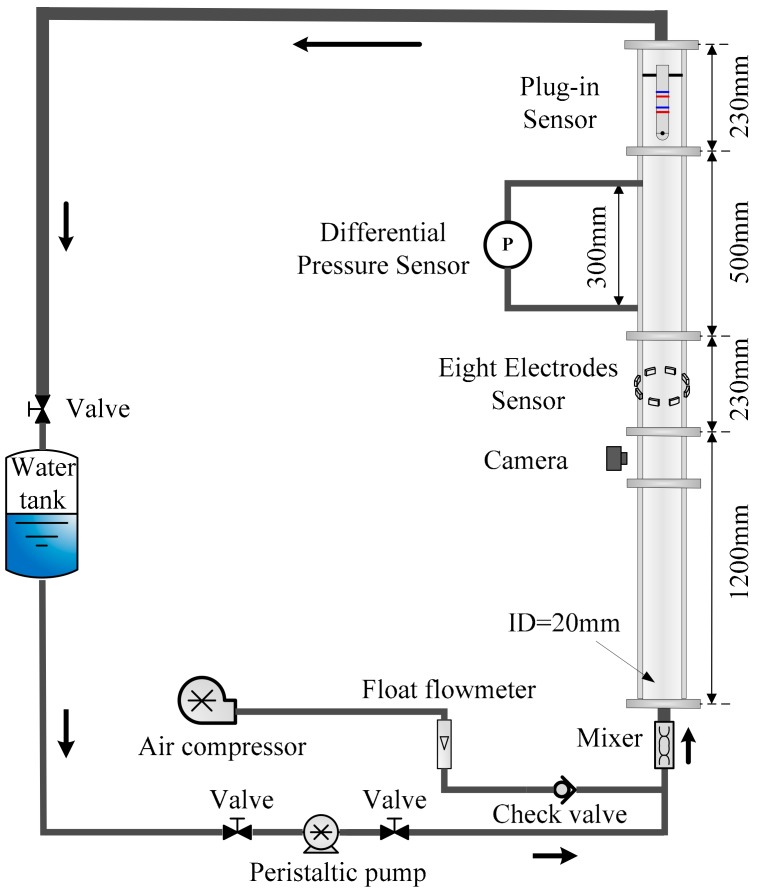
The schematic map of gas-water two-phase flow loop facility.

The experiments in vertical upward gas-water two-phase flow were implemented in flow loop facility in Tianjin University as shown in Figure 2. The experimental pipe is made up of acrylic material. The inner and outer diameter of testing pipe are 20 mm and 30 mm. In order to ensure the fluid fully develop, two pressure ports with threads (*a* and *b*) are installed at the height of 1530 mm and 1830 mm from entrance. Afterward, the two pressure ports are jointed with the press-leading tubes to measure the pressure drop. The differential pressure sensor is produced by Honeywell (model ST 700) with measurement range and precision labeled 0–25 kPa and 0.05% FS, respectively. The response time is less than 100 ms. The press-leading tubes should be full of water during experiment. The measurement system is also consisting of an eight-electrode rotating electric field conductance sensor and a plug-in cross-correlation conductance sensor to measure water holdup and cross-correlation velocity, respectively. Besides, a high-speed camera made by Weinberger Company is applied to capture the images of flow structures. To guarantee the quality of the image, the resolution is set at 384*1300, and the interval of each frame is 25 ms.

As for experimental materials, densities of water and air are 1000 kg/m^3^ and 1.29 kg/m^3^, and the surface tension between air and water phases is 0.075 N/m. An industrial peristaltic pump with high accuracy is used to control water velocity and an air compressor is used to produce the gas phase, and gas velocity is metered by a float flowmeter. The air phase and water phase are mixed at mixer at inlet of vertical pipe. During the progress of the experiment, the water superficial velocity *U_sw_* varies from 0.0368 m/s to 1.1788 m/s, and the gas superficial velocity *U_sg_* varies from 0.0553 m/s to 0.5158 m/s. Firstly, *U_sg_* is fixed, with increasing *U_sw_*, the fluctuating signals of sensors are collected. Then change the gas superficial velocity *U_sg_* and repeat the experimental process mentioned above. The fluctuating signals are acquired by PXI-4472 synchronization data acquisition board card of NI Company, with sampling frequency of 2 kHz and sampling time of 30 s for each flow condition.

## 3. Prediction Models of Pressure Drop

### 3.1. Asheim Model

The Asheim model [22] is independent of flow patterns, but does permit the selection of empirical parameters for either bubble or slug flows. The total pressure drop in two-phase flow ${\left(dp/dL\right)}_{tp}$ can be written as the sum of elevation pressure drop ${\left(dp/dL\right)}_{e}$ and friction pressure drop ${\left(dp/dL\right)}_{f}$ components. Thus, the pressure drop ${\left(dp/dL\right)}_{tp}$ and mixture density is determined from:

(8) $${\left(dp/dL\right)}_{tp}={\left(dp/dL\right)}_{e}+{\left(dp/dL\right)}_{f}=\rho_{tp}g\sin\theta +\frac{f\rho_{tp}v_{m}^{2}}{2d},$$

(9) $$\rho_{tp}=\rho_{L}H_{L}+\rho_{g}(1-H_{L}),$$

where friction factor *f* is obtained by Moody [34] diagram, which is a function of the no-slip Reynolds number. And the water holdup is expressed as:

(10) $$H_{L}=\frac{{\left[{\left(v_{sg}+a_{1}v_{sl}-a_{2}\right)}^{2}+4a_{1}a_{2}v_{sl}\right]}^{0.5}}{2a_{2}}-\frac{v_{sg}+a_{1}v_{sl}-a_{2}}{2a_{2}},$$

where *a*_1_, *a*_2_ are the empirical parameters identified by flow patterns. Asheim showed that field data can be optimized and fitted by selecting empirical parameters.

### 3.2. Hasan-Kabir Model

Hasan-Kabir [26] developed a mechanistic model to predict pressure drop. They improved the model to calculate pressure drop by considering gas-water two-phase flow as homogeneous flow, and discussed the pressure drop inside pipe based on different flow patterns. The total pressure drop in two-phase flow ${\left(dp/dL\right)}_{tp}$ can be written as Equations (8) and (9) in bubble flow.

The expression for water holdup *H_L_* is:

(11) $$H_{L}=1-\frac{v_{sg}}{C_{0}v_{m}+v_{\infty }},$$

where *C*_0_ is the flow coefficient given by different flow patterns, and $v_{\infty }$ is bubble slip or ascending velocity [35]. The friction factor *f* can be determined by the Moody [34] diagram, which is a function of the liquid phase Reynolds number, regardless of surface roughness.

While in slug flow or churn flows, due to the presence of liquid film around the Taylor bubble, the liquid film falling down along the pipe wall is opposite to the flow direction of liquid slug. Wallis [36] suggested that wall shear stress around the vapor bubble should be ignored, so in these two flow patterns, the frictional pressure drop is different from bubble flow and is expressed as:

(12) $${\left(dp/dL\right)}_{f}=\frac{f\rho_{L}v_{m}^{2}}{2d}H_{L}.$$

### 3.3. Ansari et al. Model

Ansari et al. [27] studied the mechanism and characteristics of flow patterns and established flow characteristics analysis models to describe bubble flow and slug flow. Compared to Hasan-Kabir’s model, Ansari’s model refined prediction accuracy of slug flow by considering the fully developed Taylor bubble and the developing Taylor bubble in slug flow. This study only considers the developed Taylor bubble slug flow.

For bubble flow, the slippage is considered by taking into account the bubble-rise velocity relative to mixture velocity. The actual holdup for bubble flow was expressed by an implicit equation [37] as Equation (13):

(13) 1.53 [gσL(ρL−ρg)ρL2]14HL0.5=vsg1−HL−1.2vm.

The two-phase pressure drop is the same as Equation (8), while the friction factor *f* is obtained from a Moody [34] diagram which is a function of Reynolds number under gas-liquid two phase flow.

**Figure 3:**
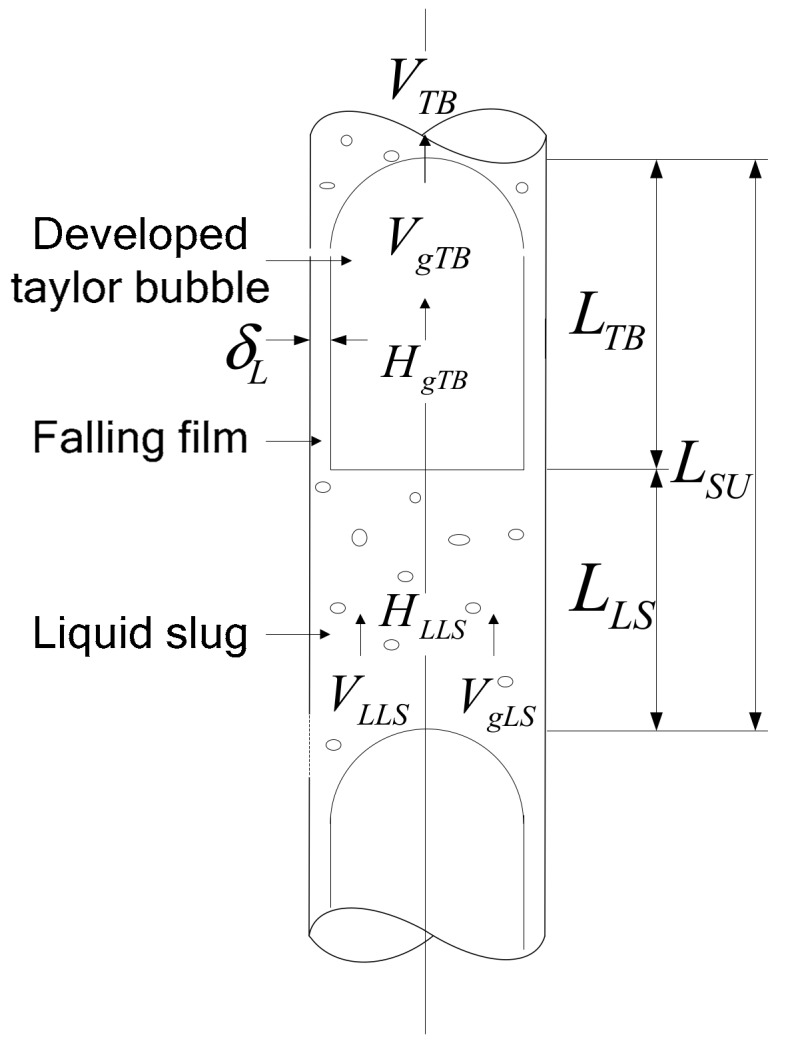
Schematic of developed slug unit.

For developed slug flow as shown in Figure 3, the elevation component occurring across a slug unit is given by:

(14) $${\left(dp/dL\right)}_{e}=\left[\left(1-\beta \right)\rho_{LS}+\beta \rho_{g}\right]g\sin\theta ,$$

where $\rho_{LS}=\rho_{L}H_{LLS}+\rho_{g}\left(1-H_{LLS}\right)$, $\beta =L_{TB}/L_{SU}$.

Ansari et al. considered that friction in developed slug flow occurs only across the liquid slug. This is given as:

(15) $${\left(dp/dL\right)}_{f}=\frac{f_{LS}\rho_{LS}v_{m}^{2}}{2d}\left(1-\beta \right).$$

### 3.4. Zhang et al. Model

The Zhang et al. model [28,29] for slug liquid holdup was based on a balance between the turbulent kinetic energy of the liquid phase and the surface free energy of dispersed spherical gas bubble. The turbulence in a liquid slug is maintained not only by the shear (or Reynolds stress) between the fluids and the pipe wall, but also by the mixing (or momentum exchange) between the slug body and the film zone of a slug unit. Therefore, the pressure gradient includes both the shear term and the mixing term.

(16) $${\left(dp/dL\right)}_{sm}={\left(dp/dL\right)}_{s}+{\left(dp/dL\right)}_{m}$$

The pressure gradient due to momentum exchange between the slug body and the film zone can be expressed as [28]:

(17) $${\left(dp/dL\right)}_{m}=\frac{\rho_{L}H_{Lf}\left(v_{t}-v_{f}\right)\left(v_{m}-v_{f}\right)}{l_{LS}},$$

where *H_Lf_* is the liquid holdup in the film zone of a slug unit. The *v_t_* is the translational velocity at which the slug unit is traveling, and *v_f_* is the liquid velocity in the film zone.

The shear stress at the pipe wall $\tau_{s}$ can be expressed as:

(18) $$\tau_{s}=\frac{f}{2}\rho_{tp}v_{m}^{2}={\left(\frac{dp}{dL}\right)}_{s}\frac{d}{4},$$

where ${\left(dp/dL\right)}_{s}$ is the pressure gradient in the slug body due to the shear stress.

### 3.5. Dynamic Two-Fluid Model

The two-fluid model is based on the conservation of mass and momentum of gas-liquid two-phase [30,31]. Two kinds of fluids coexist, which can be understood as that in a finite space element body, two kinds of fluids occupy part of the volume respectively. It can also be understood as a spatial position in the two kinds of fluid with a certain probability. Gas-liquid two-phase mass conservation equation:

(19) $$A\frac{\partial }{\partial_{t}}\left(\lambda \rho_{g}\right)+\frac{\partial }{\partial_{x}}\left(A\lambda \rho_{g}v_{sg}\right)=0,$$

(20) $$A\frac{\partial }{\partial_{t}}\left(\left(1-\lambda \right)\rho_{L}\right)+\frac{\partial }{\partial_{x}}\left(A\left(1-\lambda \right)\rho_{L}v_{sw}\right)=0.$$

Momentum conservation equation of gas-liquid two-phase:

(21) $$A\frac{\partial }{\partial_{t}}\left(\lambda \rho_{g}v_{sg}\right)+\frac{\partial }{\partial_{x}}\left(A\lambda \rho_{g}v_{sg}^{2}\right)=-A\lambda \frac{\partial_{P_{g}}}{\partial_{x}}-A\lambda \rho_{g}g\sin\theta +\Gamma_{gW}+\Gamma_{gi},$$

(22) $$A\frac{\partial }{\partial_{t}}\left(\left(1-\lambda \right)\rho_{L}v_{sw}\right)+\frac{\partial }{\partial_{x}}\left(A\left(1-\lambda \right)\rho_{L}v_{sw}^{2}\right)=-A\left(1-\lambda \right)\frac{\partial_{P_{L}}}{\partial_{x}}-A\left(1-\lambda \right)\rho_{L}g\sin\theta +\Gamma_{LW}+\Gamma_{Li}.$$

In order to achieve the purpose of the closed control equation, it is assumed that the interphase force is the interphase friction force, and the friction force on the liquid phase per unit length in the liquid slug is defined as:

(23) $$\Gamma_{Li}=\frac{1}{2}C_{D}\rho_{L}\left|v_{r}\right|v_{r}\frac{\varepsilon }{L_{SU}}A,$$

where *C_D_* is drag coefficient. The force acting on the interface by the liquid phase and the force acting on the interface by the gas phase are equal and opposite to each other:

(24) $$\Gamma_{Li}=-\Gamma_{gi}.$$

The wall shear force comes from the liquid plug area and the air plug area, then the wall shear force per unit length can be expressed as:

(25) $$\Gamma_{kW}=\tau_{k}S_{k}=\tau_{ks}\frac{l_{LS}}{l}+\tau_{kf}S_{kf}\frac{l_{TB}}{l}.$$

## 4. Results and Discussion

### 4.1. Flow Pattern Identification

In this study, the flow images acquired by the high-speed camera are used to identify gas-water two-phase flow patterns. For a primitive time series $\left\{x_{1},x_{2},\cdots ,x_{n}\right\}$, according to Takens embedding theorem (embedded dimension *m*, delay time *τ*), the space vector after the phase space reconstruction is:

(26) $$X_{i}=\left\{x_{i},x_{i+\tau },\cdots ,x_{i+\left(m-1\right)\tau }\right\}\;\;\;\;\;\;\left(i=1,2,\cdots ,N\right),$$

where *N = n − (m − 1) τ*. Then the distance between any two vectors *d_ij_* in these vector sets is:

(27) $$d_{ij}=\left\|X_{i}-Y_{j}\right\|.$$

Select the threshold value *r*, then the recursive matrix *R_ij_ = Heaviside(r − d_ij_)* can be obtained, where the *Heaviside* function is expressed as follows:

(28) $$Heaviside\left(x\right)=\left\{\begin{array}{c} 1\;\;\;\;x\geq 0\\ 0\;\;\;x<0 \end{array}\right..$$

Based on the result of *R_ij_*, points were drawn on the coordinate plane with the number of time series on the vertical axis and the horizontal axis. *R_ij_* = 1 denotes the occurrence of recursion, and a black dot is drawn on the corresponding position on the plane. *R_ij_* = 0 means that the recursion does not occur, then no point will be drawn at the corresponding position of the recursion graph. Accordingly, the corresponding recursive texture structure can be drawn. In this study, delay time *τ* = 2, phase space dimension *m* = 1, and empirical coefficient *α* = 0.25 [38] are set. Because the conductance sensor is applied with the superiority of quick response, the recurrence plot of REFCS signals under different conditions are analyzed.

**Figure 4:**
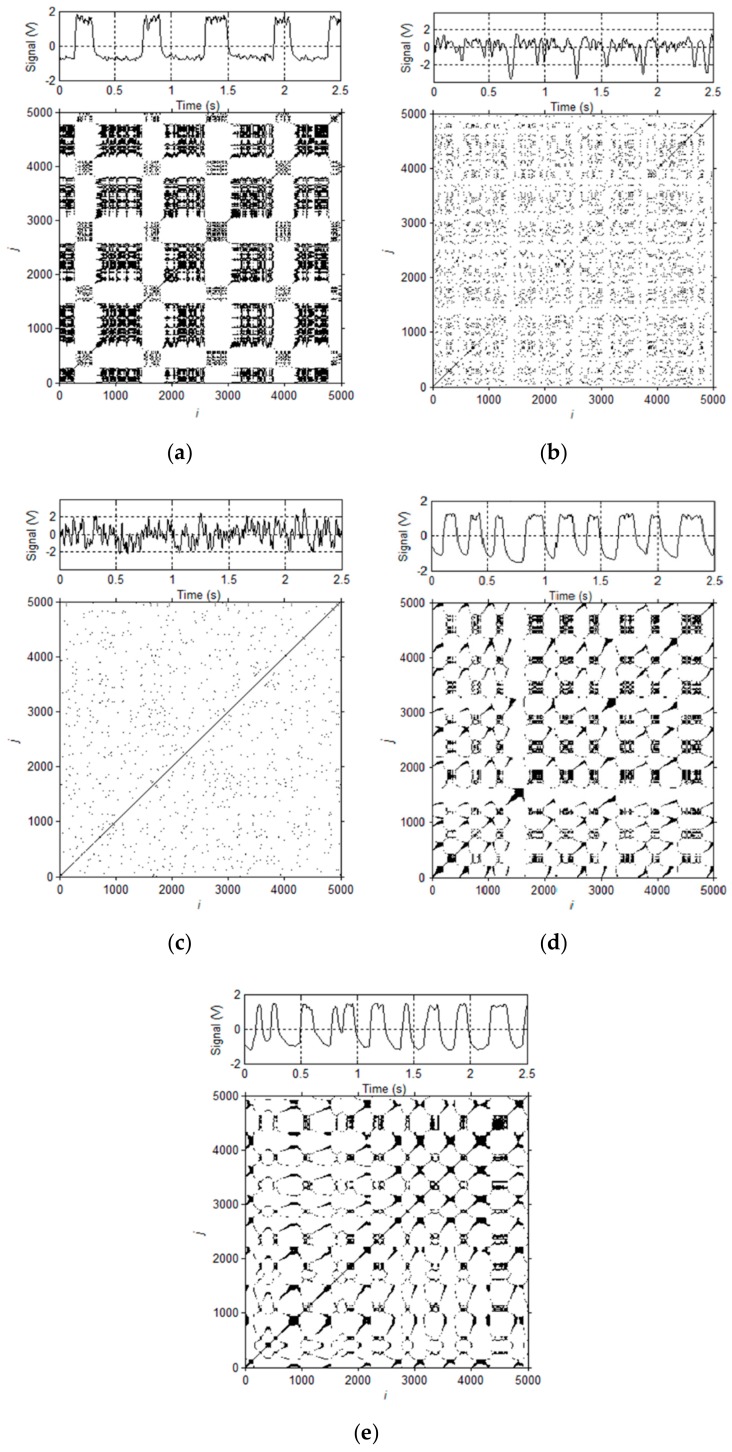
Recurrence plots of different flow patterns. (**a**) Slug flow (*U_sg_* = 0.0552 m/s, *U_sw_* = 0.0368 m/s); (**b**) Bubble-slug transitional flow (*U_sg_* = 0.0552 m/s, *U_sw_* = 0.5888 m/s); (**c**) Bubble flow (*U_sg_* = 0.0552 m/s, *U_sw_* = 1.1776 m/s); (**d**) Slug-churn transitional flow (*U_sg_* = 0.4416 m/s, *U_sw_* = 0.5888 m/s); (**e**) Churn flow (*U_sg_* = 0.4416 m/s, *U_sw_* = 1.1776 m/s).

**Figure 5:**
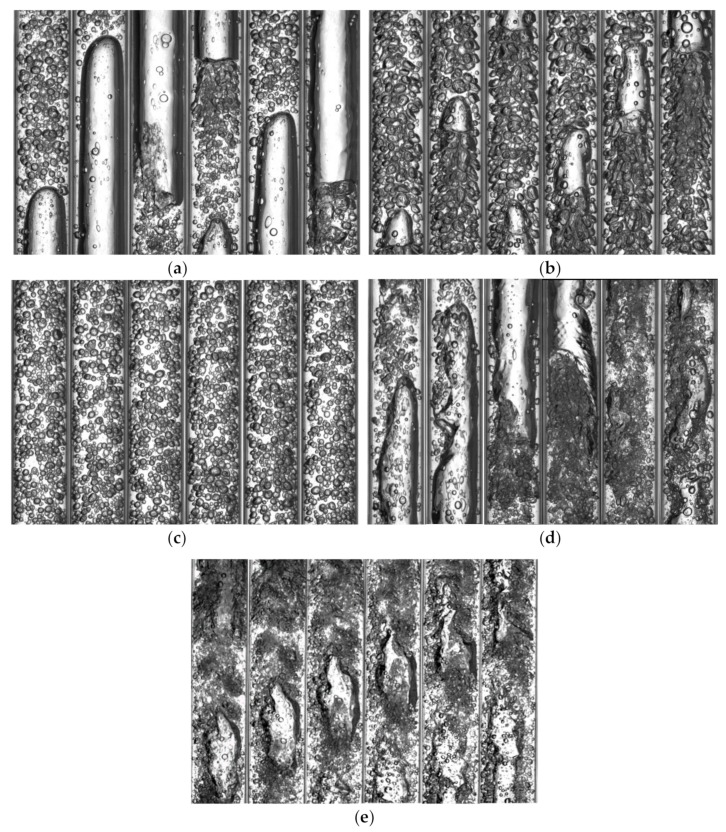
Visualizations of experimental flow patterns, and the interval of each frame is 25 ms. (**a**) Slug flow (*U_sg_* = 0.0552 m/s, *U_sw_* = 0.0368 m/s); (**b**) Bubble-slug transitional flow (*U_sg_* = 0.0552 m/s, *U_sw_* = 0.5888 m/s); (**c**) Bubble flow (*U_sg_* = 0.0552 m/s, *U_sw_* = 1.1776 m/s); (**d**) Slug-churn transitional flow (*U_sg_* = 0.4416 m/s, *U_sw_* = 0.5888 m/s); (**e**) Churn flow (*U_sg_* = 0.4416 m/s, *U_sw_* = 1.1776 m/s).

The recurrence plots of slug flow, slug-bubble transition flow, bubble flow, slug-churn transition flow and churn flow are shown in Figure 4a–e, respectively, and the corresponding visualizations of experimental flow patterns are shown in Figure 5a–e. The flow characteristic of slug flow is that there are obvious periodic alternating motions of Taylor bubble and liquid slug as shown in Figure 5a, which corresponds to obvious line texture and intermittent rectangular structures existing in recurrence plot and developing along the diagonal direction as shown in Figure 4a. As the velocity of water phase increases, slug flow gradually evolves into bubbly flow as shown in Figure 5b. The Taylor bubble is crushed, but there are still a small number of gas blocks. The flow characteristics have a certain degree of periodic motion characteristics which are similar to the slug flow. The block texture structure is faintly visible, but the recurrence plot mainly presents a line structure with poor development along the diagonal direction and a large number of scattered points as shown in Figure 4b, which proves that there are a certain number of small bubbles.

Due to the huge turbulent energy in the pipeline, the Taylor bubble is crushed completely to form bubble flow as presented in Figure 5c, which is approximately evenly distributed in the water. The conductance fluctuation signal of bubble flow is similar to random noise, so the recurrence plot of bubble flow has obvious irregularities and scatters, and exhibits similar random dotted structure features as shown in Figure 4c. As the velocity of gas phase increases, it can be seen that the flow pattern presents the periodic motion characteristics like the slug flow, and a large block gas plug still existed as shown in Figure 5d. The transitional flow pattern has the characteristics of two typical flow patterns as presented in Figure 4d. There are still black rectangular texture structures in the recurrence plot which can reflect the flow characteristics of the slug flow. As the Taylor bubble is out of shape, the recurrence plot shows that the black rectangular blocks become small and the texture lines appear.

The churn flow is a flow pattern in which the gas is mixed with a continuous liquid phase with a higher turbulent energy when the Taylor bubble in the slug flow is crushed as shown in Figure 5e. As shown in Figure 4e, the black block structures nearly disappear, showing the uniform and dispersed structure characteristic. This can be attributed to the fact that this flow pattern exhibits extremely unstable oscillation characteristics. The violent oscillation causes the gas block to pass through the sensor quickly, which cause a short texture structure in the recurrence plot.

**Figure 6:**
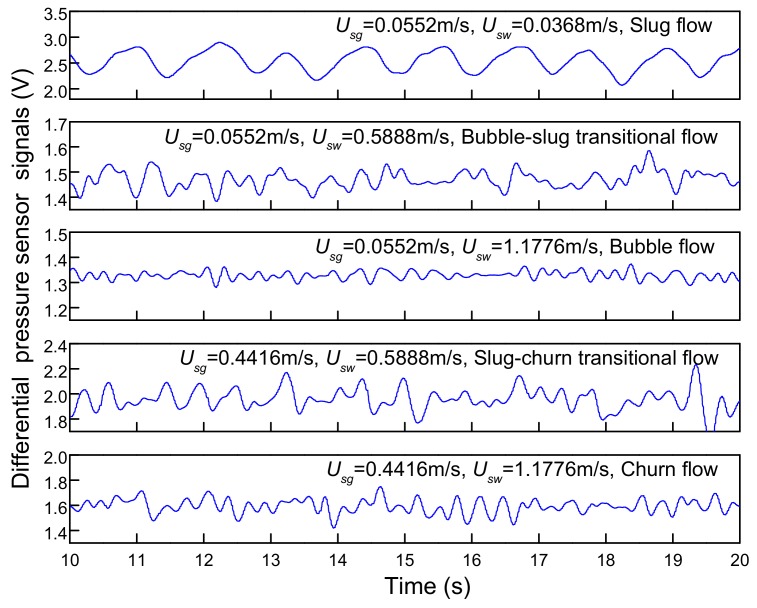
The responses of differential pressure sensor.

**Figure 7:**
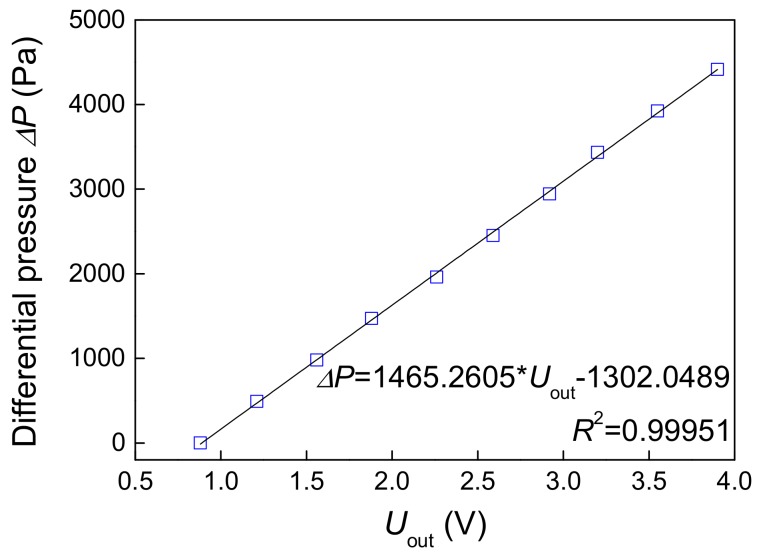
Calibration result of differential pressure sensor.

The response of differential pressure sensor of five flow patterns are given in Figure 6. The voltage signal can reflect the pressure fluctuation under different flow patterns. For slug flow, the turbulent energy of fluid is relatively low, and the Taylor bubble in slug flow is not easy to be broken into small gas blocks. As these Taylor bubbles pass through differential pressure sensor, the density of fluid experiences has a great change, resulting in an apparent fluctuation on output of differential pressure sensor. When flow pattern evolves to churn flow, turbulent energy increases and the Taylor bubble is broken up into small gas blocks, which leads to unobvious variations of fluid density. Therefore, the output of differential pressure sensor fluctuates with small amplitude and high frequency. As for bubble flow, the turbulent energy becomes very large and gas bubbles become small with higher velocity. The output of differential pressure sensor demonstrates fluctuations of the smallest amplitude and highest frequency in all flow patterns. As illustrated in Figure 6, it can be inferred that small discrepancies of differential pressure sensor outputs under different flow conditions will induce the difficulty of distinguishing foregoing flow patterns especially in transitional flow. It can be attributed to the low response frequency of differential pressure sensor, which makes it insensitive to the variation of flow structures. Then, the voltage signal output by differential pressure sensor is converted into differential pressure value through static calibration, and the calibration result is shown as Figure 7. The results show that there is a good linear relationship between *U_out_* and Δ*P*. Then the subsequent calculation can be carried out according to the differential pressure after voltage conversion.

**Figure 8:**
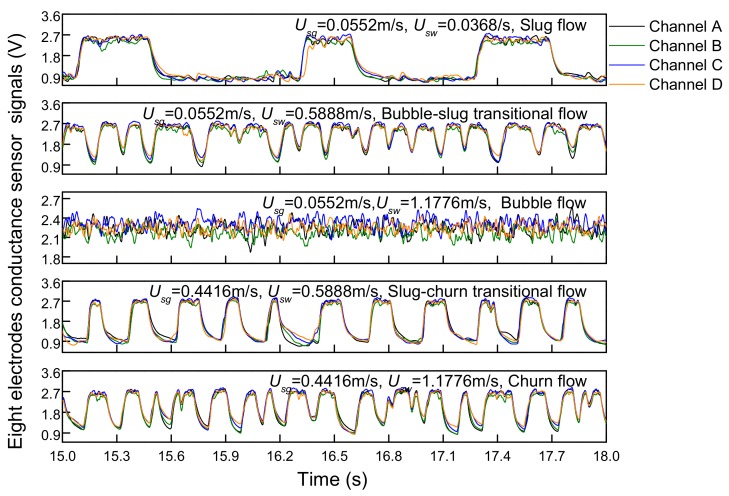
The responses of an eight-electrode rotating electric field conductance sensor.

**Figure 9:**
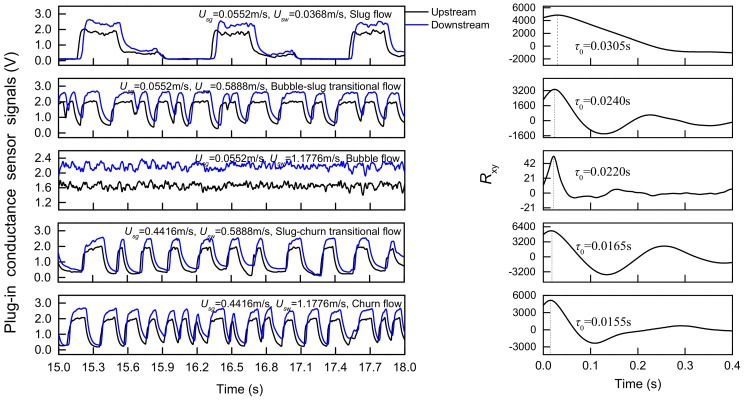
The responses of plug-in cross-correlation conductance sensor and correlation function analysis.

The fluctuation signals of an eight-electrode rotating electric field conductance sensor are shown in Figure 8. In slug flow, the high voltage signal indicates that liquid slug passes through the measurement area, and the low voltage signal indicates that Taylor bubble passes through the measurement area. The liquid slug and Taylor bubble alternately passed through the sensor, which makes the signal present significantly periodic characteristics. When the water superficial velocity becomes higher, the flow pattern evolves to bubble-slug transitional flow with large gas blocks broken into small bubbles, but signal occasionally shows a low voltage, which means there are still gas blocks passing by. The signal amplitude fluctuation of bubble flow is concentrated in a small range, reflecting the random and non-uniform distribution of small bubbles. When the flow pattern changes from the slug flow to the churn flow, sensor signals also have periodic characteristics, but the durations of high and low voltage are shorter than that in slug flow. Additionally, higher turbulent energy leads to stronger fluctuation. In churn flow, the increasing flow velocity make Taylor bubble break and deform. Taylor bubble mixes with the liquid slug to form a chaotic and disordered mixture. Therefore, the frequency of signal fluctuation increases, and the signal fluctuation is more irregular. The sensor responses of plug-in conductance sensors are shown in Figure 9. It can be seen that the upstream and downstream signals have good correlation and the cross-correlation functions present obvious peaks, which can be ascribed to the fact that the center body increases the flow velocity to make the flow structures stable in the annular space and thus enhances the correlation of upstream and downstream signals. The time corresponding to the peak value is the delay time *τ_0_* in Figure 8, then the cross-correlation velocity *U_cc_* can be calculated according to Formula (7). Besides, plug-in conductance sensor can also reflect flow characteristics under different flow patterns similar to REFCS response.

### 4.2. Model Test

Five models are used to predict the pressure drop of gas-water two-phase flow in this study, and compared with the pressure drop measured by differential pressure sensor which converted by voltage signal. Among them, the flow pattern identification is determined according to the recurrence plot method.

For evaluating the predication results, three evaluation criterions are introduced, named average percentage deviation (APD), absolute average percentage deviation (AAPD), and root mean square percentage deviation (RMSPD), which can be calculated through the expressions below:

(29) $$\mathrm{APD}=\frac{1}{n}\;\sum_{k=1}^{n}\;\left[\frac{{\left(dp/dz\right)}_{pre}-{\left(dp/dz\right)}_{\exp}}{{\left(dp/dz\right)}_{\exp}}\right]\times 100\%,$$

(30) $$\mathrm{AAPD}=\frac{1}{n}\;\sum_{k=1}^{n}\left|\frac{{\left(dp/dz\right)}_{pre}-{\left(dp/dz\right)}_{\exp}}{{\left(dp/dz\right)}_{\exp}}\right|\times 100\%,$$

(31) $$\mathrm{RMSPD}=\sqrt{\frac{1}{n}\;\sum_{k=1}^{n}\;{\left[\frac{{\left(dp/dz\right)}_{pre}-{\left(dp/dz\right)}_{\exp}}{{\left(dp/dz\right)}_{\exp}}\right]}^{2}}\times 100\%.$$

**Figure 10:**
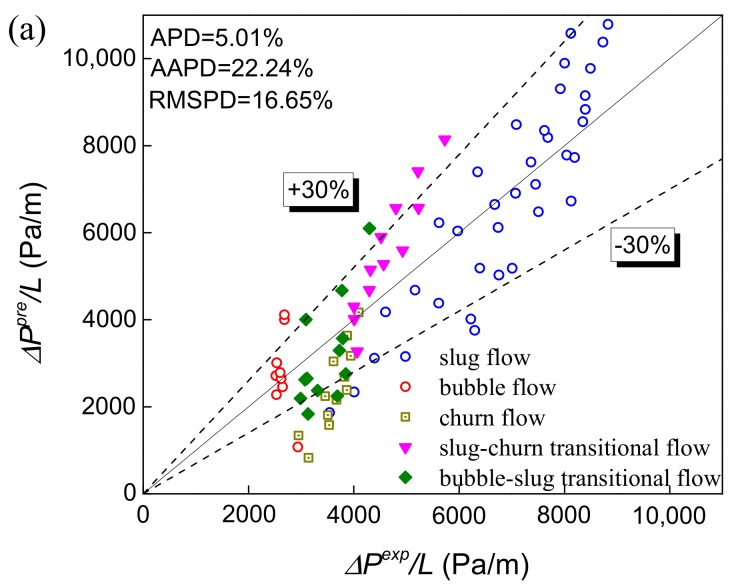

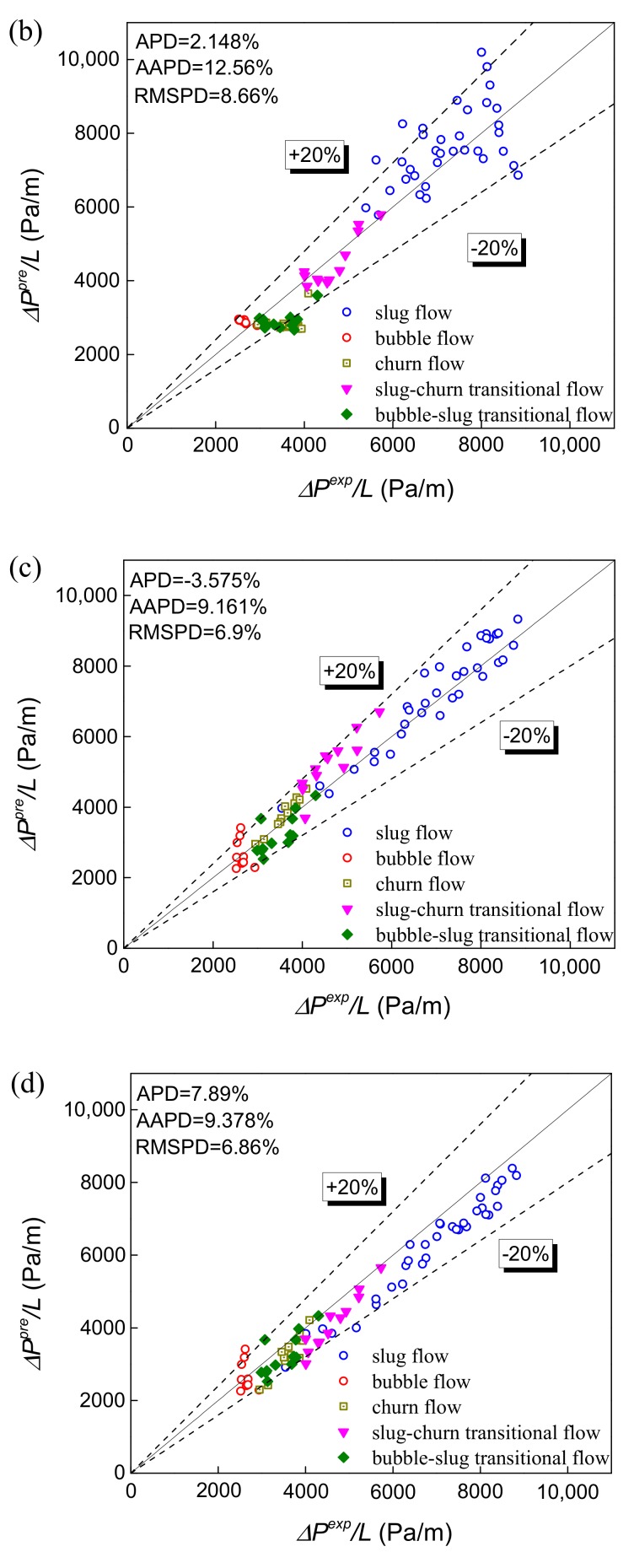

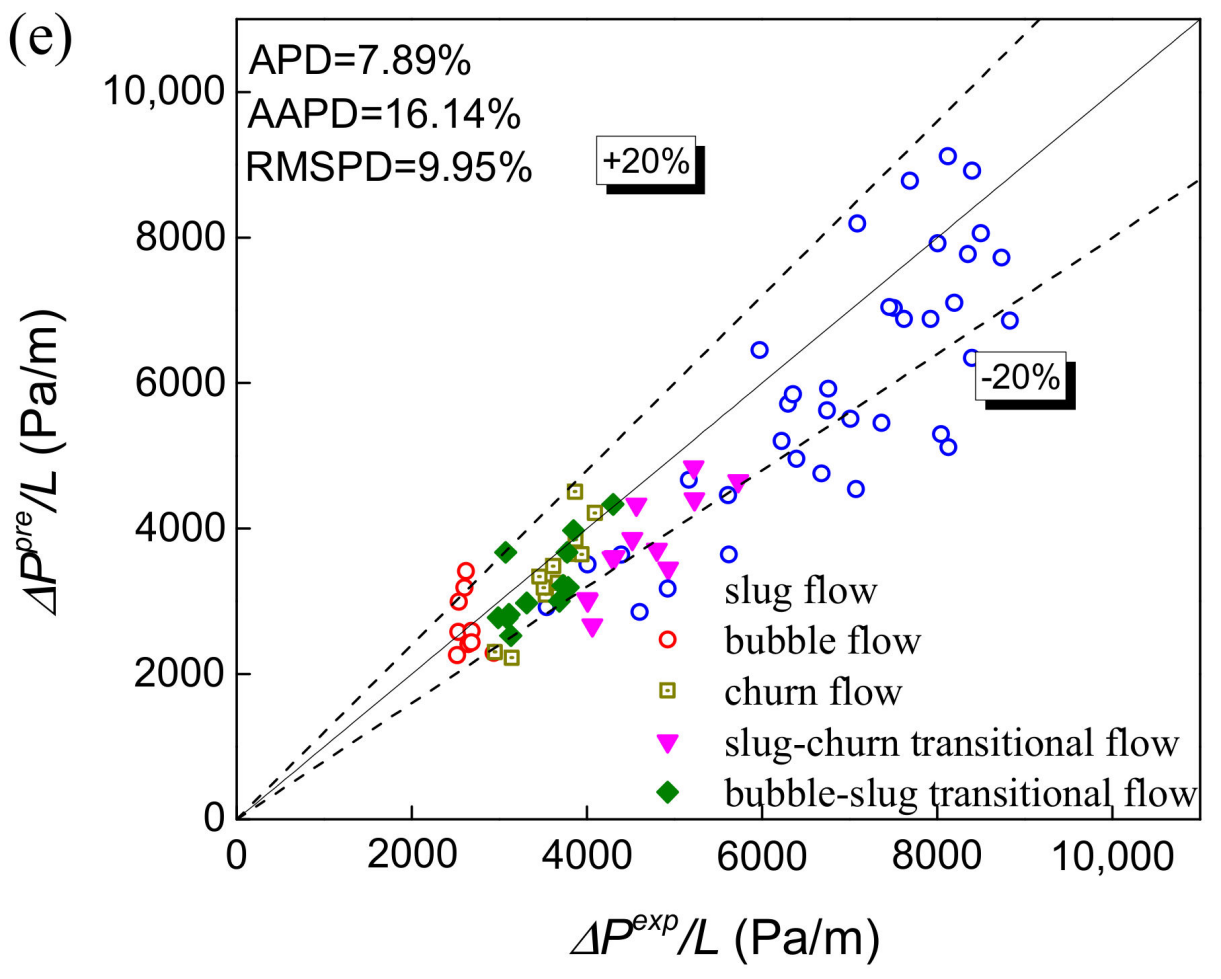
The prediction accuracy of pressure drop. (**a**) Asheim model; (**b**) Hasan-Kabir model; (**c**) Ansari et al. model. (**d**) Zhang et al. model; (**e**) Dynamic two-fluid model.

The gas-water two-phase flow pressure drop gradient prediction and three evaluation criterions are shown in Figure 10a–e. Churn flow has not yet been modeled because of its complexity and it is treated as part of slug flow. Through high-speed images, it can be found that the slug-bubble transitional flow has a certain similarity with bubble flow. Therefore, the slug-bubble transition flow is calculated according to the bubble flow model. Besides, the churn flow and slug-churn transition flow are calculated according to the slug flow model in this study.

The prediction results using Asheim model [22] are shown in Figure 10a. This model only uses fixed empirical parameters to calculate the pressure drop without considering the influence of the flow pattern characteristics to the fluid. However, the influence of slug length on pressure drop in slug flow and the complexity of two transitional flow and churn flow will lead to the partial error of pressure drop prediction. Therefore, the accuracy of pressure drop prediction is low with AAPD equaling 22.24%. As shown in Figure 10b, the prediction results using the Hasan-Kabir model [26] which predict pressure drop considering the flow mechanism and flow pattern distribution. The accuracy is greatly improved by considering the different influences of Taylor bubble and liquid slug on the frictional pressure drop compared with Asheim’s prediction model, and the AAPD drops to 12.56%. However, this model only uses the homogeneous Reynolds number which still leads to deviation of pressure drop prediction in slug flow.

The prediction results using Ansari et al. model [27] are shown in Figure 10c. The accuracy of this model has been improved obviously with the AAPD equaling 9.161%. The pressure drop prediction accuracy is higher in slug flow by considering the length of the liquid slug and Taylor bubble, and calculating the gravity pressure drop and friction pressure drop, respectively. Meanwhile, the Ansari et al. model determines the friction factor based on two-phase flow mixture Reynolds number instead of the homogeneous Reynolds number, and further improves the pressure drop prediction accuracy. However, the prediction accuracy of transitional flow patterns is lower compared to other flow patterns, due to its complex flow structure. Overall, the prediction accuracy of the Ansari et al. model is better.

The pressure drop prediction results of the Zhang et al. model [28,29] and the two-fluid model [30,31] are presented in Figure 10d,e, respectively. These two models are more targeted at slug flow analysis, so the slug flow is calculated by them and churn flow, bubble flow and their transition flow patterns are predicted by the Ansari model in this paper. The pressure drop prediction of the Zhang et al. model is good, but there is still some systematic deviation. And the model needs to measure the liquid film velocity, which is not easy to measure directly in industrial application. The two-fluid model still has problems in the construction of the correlation formula to describe the interaction between the fluid and the wall surface and the phase interface, resulting in poor prediction. Besides, the two-fluid model is more used in the case of horizontal flow and stratified flow. The ability to predict the pressure drop of vertical rising slug flow needs to be improved.

## 5. Pressure Drop Prediction

Comparing the prediction results of the three models, the Ansari et al. model can predict the pressure drop in vertical upward gas-water two-phase flow with higher accuracy. Therefore, the dynamic experiment was carried out to obtain relevant parameters required for pressure drop prediction in the Ansari et al. model. Thereby, for improving prediction accuracy, a sensor combination method for predicting pressure drop is established. Firstly, the expression of friction factor derived from the Ansari et al. model and Equation (2) is as follows:

(32) $$\Delta P=\left\{\begin{array}{l} \rho_{y}gh-\left[\rho_{tp}gh+\frac{f_{tp}\rho_{tp}v_{tp}^{2}}{2d}h\right]\;\;\;\;\;\;\left(\mathrm{bubble}\;\mathrm{flow}\right)\\ \rho_{y}gh-\left\{\left[\left(1-\beta \right)\rho_{LS}+\beta \rho_{g}\right]gh+\frac{f_{LS}\rho_{LS}v_{m}^{2}}{2d}\left(1-\beta \right)h\right\}\;\;\left(\mathrm{slug}\;\mathrm{flow}\right) \end{array}\right.,$$

(33) $$f_{}=\left\{\begin{array}{l} \frac{(\Delta P-\rho_{y}gh-\rho_{tp}gh)*2d}{\rho_{tp}v_{tp}^{2}h}\;\;\;\;\;\;\left(\mathrm{bubble}\;\mathrm{flow}\right)\\ \frac{(\Delta P-\rho_{y}gh-\left[\left(1-\beta \right)\rho_{LS}+\beta \rho_{g}\right]gh)*2d}{\rho_{LS}v_{m}^{2}h\left(1-\beta \right)}\;\;\;\left(\mathrm{slug}\;\mathrm{flow}\right) \end{array}\right..$$

**Figure 11:**
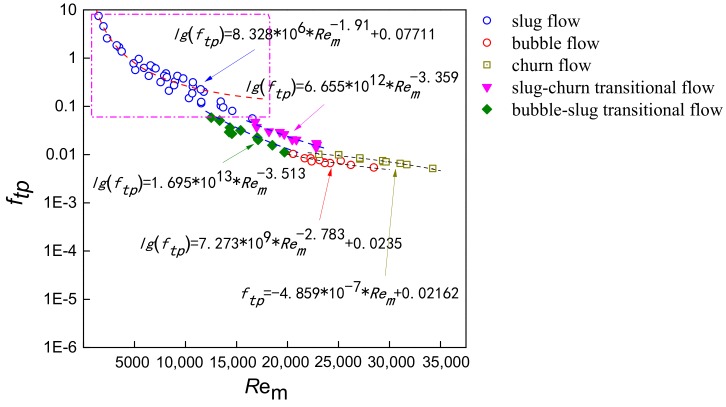
Relationships between experimental friction factor *f_tp_* and mixture Reynolds number *R_em_*.

Then, the mixture Reynolds number is determined based on the water holdup measured by REFCS, and the friction factor curve is fitted. The bubble flow and the slug-bubble transition flow pattern are calculated as bubble flow. The slug flow, the churn flow and the slug-churn transition flow are calculated as slug flow. The differential pressure sensor measures the fluid pressure drop within a 300 mm height, and the plug-in conductance sensor obtain the cross-correlation velocity and slug unit length. Meanwhile, the eight-electrode rotating electric field conductance sensor measures the water holdup. For different flow patterns, the fitting curves of mixture friction factor and mixture Reynolds number are shown in Figure 11 and expressed as follows:

(34) $$\left\{\begin{array}{l} lg\left(f_{tp}\right)=8.328*10^{6}*Re_{m}^{-1.91}+0.07711\;\;(\mathrm{slug}\;\mathrm{flow})\\ lg\left(f_{tp}\right)=7.273*10^{9}*Re_{m}^{-2.783}+0.0235\;\;(\mathrm{bubble}\;\mathrm{flow})\\ f_{tp}=-4.859*10^{-7}*Re_{m}^{}+0.02162\;\;\;(\mathrm{churn}\;\mathrm{flow})\\ lg\left(f_{tp}\right)=6.655*10^{12}*Re_{m}^{-3.359}\;\;\;\;(\mathrm{slug}\text{-}\mathrm{churn}\;\mathrm{transitional}\;\mathrm{flow})\\ lg\left(f_{tp}\right)=1.695*10^{13}*Re_{m}^{-3.513}\;\;\;\;(\mathrm{bubble}\text{-}\mathrm{slug}\;\mathrm{transitional}\;\mathrm{flow}) \end{array}\right..$$

It can be seen from Figure 11 that under the slug flow, the friction factor of two-phase flow is large and the distribution is more dispersed, as slug flow presents the most obvious slippage between phases and the mixture fluid possesses great instability. In addition, the transition trend of two transitional flows can be clearly seen, indicating that flow patterns are accurately recognized. As the water superficial velocity increases, the stability of mixture fluid strengthens. Hence, *f_tp_* declines acutely with the increasing Reynolds number, and the flow pattern evolves to bubble flow. For bubble flow, as the distribution of dispersed phase becomes more homogeneous due to the enhancing turbulence energy, the value of *f_tp_* shows the weakest fluctuation.

**Figure 12:**
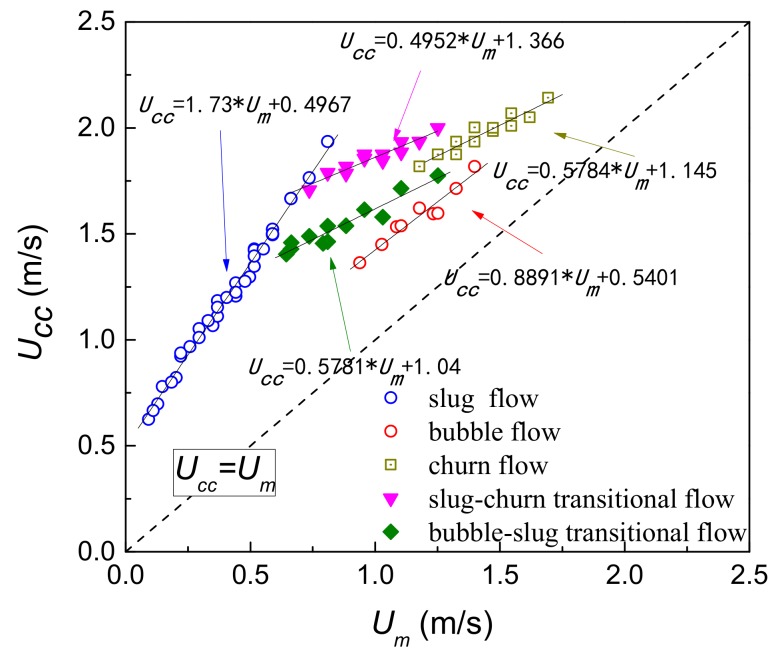
Relationships between mixture velocity *U_m_* and cross-correlation velocity *U_cc_*.

According to the cross-correlation velocity measurement principle, the relationship between mixture velocity *U_m_* and cross-correlation velocity *U_cc_* is determined experimentally, as shown in Figure 12. The mixture velocity can be determined by measuring cross-correlation velocity. And combined with the measurement time, the length of Taylor bubble and liquid slug could be calculated. It can be seen from Figure 11 that the linearity of the relationships is high under different flow patterns, and the relationships between *U_m_* and *U_cc_* can be expressed as follows:

(35) $$U_{cc}=\left\{\begin{array}{l} 1.73*U_{m}+0.4967\;\;\;\;\left(\mathrm{slug}\;\mathrm{flow}\right)\\ 0.8891*U_{m}+0.5401\;\;\;\;\left(\mathrm{bubble}\;\mathrm{flow}\right)\\ 0.5784*U_{m}+1.145\;\;\;\;\left(\mathrm{churn}\;\mathrm{flow}\right)\\ 0.4952*U_{m}+1.366\;\;\;\;\left(\mathrm{slug}\text{-}\mathrm{churn}\;\mathrm{transitional}\;\mathrm{flow}\right)\\ 0.5781*U_{m}+1.04\;\;\;\;\left(\mathrm{bubble}\text{-}\mathrm{slug}\;\mathrm{transitional}\;\mathrm{flow}\right) \end{array}\right.,$$

**Figure 13:**
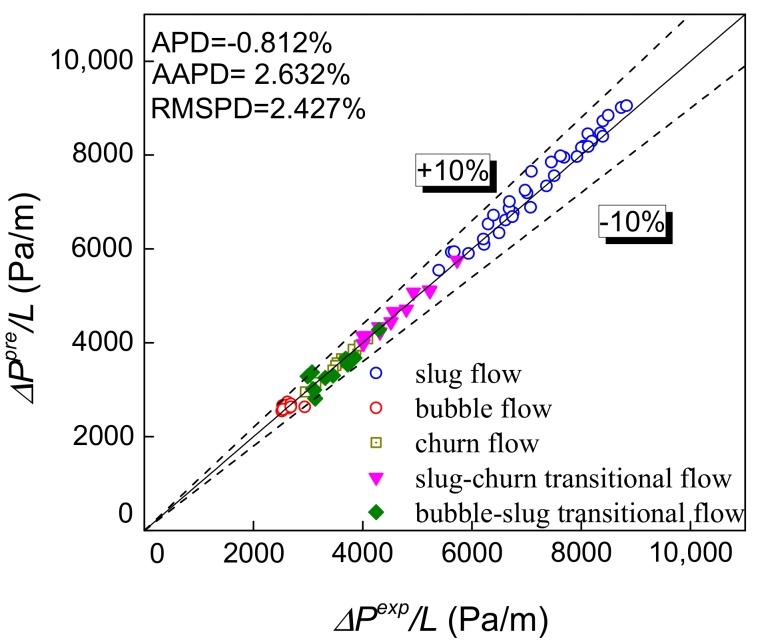
Comparison of pressure drop prediction and experimental results.

**Figure 14:**
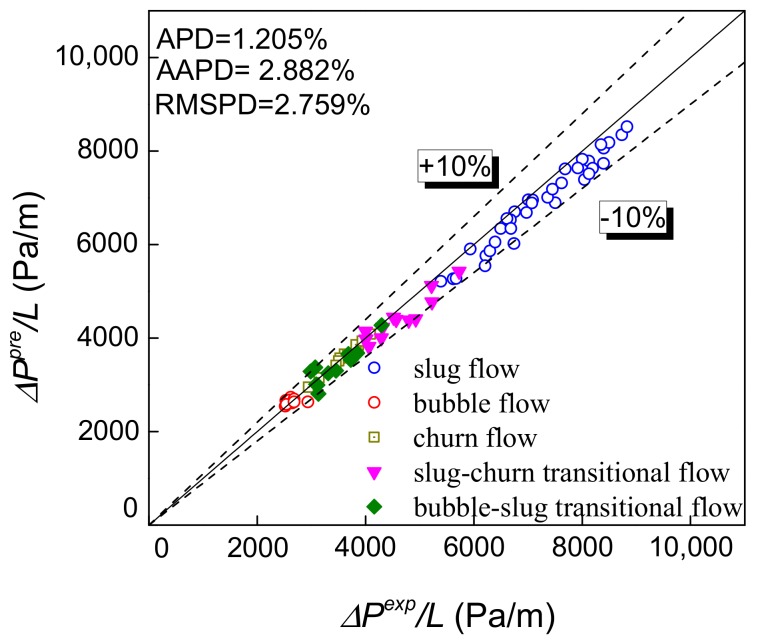
Pressure drop prediction of Zhang et al. model modified by using mixture friction factor.

Based on the measured velocity, the length of Taylor bubble and slug unit are obtained, thereby the ratio *β* of *L_TB_* and *L_SU_* can be calculated. The REFCS obtains the water holdup to determine the mixture density. All of the above parameters are substituted into Equation (30), so the predicted pressure drop in gas-water two-phase flow can be obtained as shown in Figure 13. It can be seen that based on Ansari et al. model and the measured inlet parameters obtained by the combination of pressure-conductance sensor designed in this study, the pressure drop can be predicted with high accuracy with the AAPD less than 2.632%. In addition, the pressure drop prediction of Zhang et al. model is modified by the mixture friction factor, and the prediction accuracy is improved as shown in Figure 14. It is proved that the prediction effect by using the mixture friction factor obtained through the combination of differential pressure and conductance sensor is better than that by the liquid phase friction factor.

## 6. Conclusions

(1) In this paper, a pressure drop prediction system is designed based on a combination of an eight-electrode rotating electric field conductance sensor (REFCS), plug-in cross-correlation conductance sensor and differential pressure sensor. This combination overcomes the limitation of the existing pressure drop prediction model that the inlet flow velocity needs to be known.

(2) The upward gas-water two-phase flow in small diameter pipe experiment is carried out in the multiphase flow laboratory of Tianjin University. The response of REFCS is used to identify flow pattern and measure water holdup. During which five flow patterns are identified, i.e., slug flow, bubble flow, churn flow, bubble-slug transitional flow, and slug-churn transitional flow. Meanwhile, the mixture velocity of two-phase flow is determined by the plug-in conductance sensor. The differential pressure sensor provides differential pressure fluctuation signal. The mixture friction factor of gas-water two-phase flow is obtained by fitting method based on the measured parameters and flow pattern identification.

(3) The prediction performances of five pressure drop models are evaluated and verified in this paper. The results show that the model proposed by Ansari et al. suggests a higher pressure drop prediction accuracy compared with other four differential pressure model. Moreover, the accuracy of pressure drop prediction of the Zhang et al. model is improved by using the mixture friction factor. In conclusion, a differential pressure sensor coupled with conductance sensors for predicting pressure drop presents good performance in improving the accuracy of pressure drop. Meanwhile, the high precision model of pressure drop prediction has significant value for measuring mixture density in gas-water two phase flow.

## Nomenclature

*d*	pipe diameter
*f*	friction factor
*g*	acceleration due to gravity
*H*	average holdup fraction
*L*	length along the pipe
*v*	velocity
*β*	length ratio
*ϴ*	angle from horizontal
*λ*	no-slip gas holdup fraction
*ρ*	density
Γ	interaction force
*σ*	surface tension
**Subscripts**	
*e*	elevation
*f*	film
*g*	gas
*i*	x direction
*L*	liquid
*LS*	liquid slug
*r*	relative
*s*	shear stress
*SU*	slug unit
*tp*	two-phase
*TB*	taylor bubble
*w*	wall

## References

[1] Zhai L.S., Bian P., Han Y.F., Jin N.D. (2016). The measurement of gas–liquid two-phase flows in a small diameter pipe using a dual-sensor multi-electrode conductance probe. Meas. Sci. Technol..

[2] Gao Z.K., Yang Y., Zhai L.S., Jin N.D., Chen G. (2016). A four-sector conductance method for measuring and characterizing low-velocity oil-water two-phase flows. IEEE Trans. Instrum. Meas..

[3] Merilo M., Dechene R.L., Cichowlas W.M. (1977). Void fraction measurement with rotating electric field conductance gauge. J. Heat Trans..

[4] Rocha M.S., Simões-Moreira J.R. (2008). Void fraction measurement and signal analysis from multiple-electrode impedance sensors. Heat Transf. Eng..

[5] Zhuang L.X., Jin N.D., Zhao A., Gao Z.K., Zhai L.S., Tang Y. (2016). Nonlinear multi-scale dynamic stability of oil–gas–water three-phase flow in vertical upward pipe. Chem. Eng. J..

[6] Beck M.S. (1981). Correlation in instruments: Cross correlation flowmeters. J. Phys. E Sci. Instrum..

[7] Yan Y. (1996). Mass flow measurement of bulk solids in pneumatic pipelines. Meas. Sci. Technol..

[8] Takashima S., Asanuma H., Niitsuma H. (2004). A water flowmeter using dual fiber Bragg grating sensors and cross-correlation technique. Sens. Actuators A Phys..

[9] Saoud A., Mosorov V., Grudzien K. (2016). Measurement of velocity of gas/solid swirl flow using Electrical Capacitance Tomography and cross correlation technique. Flow Meas. Instrum..

[10] Jin H., Han Y., Yang S., He G. (2010). Electrical resistance tomography coupled with differential pressure measurement to determine phase hold-ups in gas–liquid–solid outer loop bubble column. Flow Meas. Instrum..

[11] Shafquet A., Ismail I. Measurement of void fraction by using electrical capacitance sensor and differential pressure in air-water bubble flow. Proceedings of the 2012 4th International Conference on Intelligent and Advanced Systems.

[12] Jia J., Babatunde A., Wang M. (2015). Void fraction measurement of gas–liquid two-phase flow from differential pressure. Flow Meas. Instrum..

[13] Lockhart R.W., Martinelli R.C. (1949). Proposed correlation of data for isothermal two-phase, two-component flow in pipes. Chem. Eng. Prog..

[14] Fancher J.R., Brown K.E. (1963). Prediction of pressure gradients for multiphase flow in tubing. SPE.

[15] Chisholm D. (1973). Pressure gradients due to friction during the flow of evaporating two-phase mixtures in smooth tubes and channels. Int. J. Heat Mass Transf..

[16] Müller-Steinhagen H., Heck K. (1986). A simple friction pressure drop correlation for two-phase flow in pipes. Chem. Eng. Process..

[17] Poettman F.H., Carpenter P.G. (1952). The multiphase flow of gas, oil, and water through vertical flow strings with application to the design of gas-lift installations. Drilling and Production Practice.

[18] Baxendell P.B., Thomas R. (1961). The calculation of pressure gradients in high-rate flowing wells. J. Petrol. Technol..

[19] Hagedorn A.R., Brown K.E. (1965). Experimental study of pressure gradients occurring during continuous two-phase flow in small-diameter vertical conduits. J. Petrol. Technol..

[20] Duns J.H., Ros D.J. Vertical flow of gas and liquid mixtures in wells. Proceedings of the 6th World Petroleum Congress.

[21] Chierici G.L., Ciucci G.M., Sclocchi G. (1974). Two-phase vertical flow in oil wells—Prediction of pressure drop. J. Petrol. Technol..

[22] Asheim H. (1986). An accurate two-phase well flow model based on phase slippage. SPE Prod. Eng..

[23] Sarica C., Schmidt Z., Doty D. (1999). A Mechanistic Model for Predicting Pressure Drop in Vertical Upward Two-Phase Flow. J. Energ. Resour. ASME.

[24] Abdul-Majeed G.H., Al-Mashat A.M. (2000). A mechanistic model for vertical and inclined two-phase slug flow. J. Petrol. Sci. Eng..

[25] Shiferaw D., Mahmoud M., Karayiannis T.G., Kenning D.B.R. (2011). One-dimensional semi mechanistic model for flow boiling pressure drop in small to micro passages. Heat Transf. Eng..

[26] Hasan A.R., Kabir C.S. (1990). Performance of a two-phase gas/liquid flow model in vertical wells. J. Petrol. Sci. Eng..

[27] Ansari A.M., Sylvester N.D., Sarica C., Shoham O. (1994). A comprehensive mechanistic model for upward two-phase flow. SPE Prod. Facil..

[28] Zhang H.Q., Jayawardena S.S., Redus C.L., Brill J.P. (2000). Slug Dynamics in Gas-Liquid Pipe Flow. J. Energ. Resour. ASME.

[29] Zhang H.Q., Wang Q., Sarica C., Brill J.P. (2003). A unified mechanistic model for slug liquid holdup and transition between slug and dispersed bubble flows. Int. J. Multiph. Flow.

[30] Yan K., Che D. (2011). Hydrodynamic and mass transfer characteristics of slug flow in a vertical pipe with and without dispersed small bubbles. Int. J. Multiph. Flow.

[31] Abdulkadir M., Hernandez-Perez V., Lowndes I.S., Azzopardi B.J., Dzomeku S. (2014). Experimental study of the hydrodynamic behaviour of slug flow in a vertical riser. Chem. Eng. Sci..

[32] Wang D.Y., Jin N.D., Zhuang L.X., Zhai L.S., Ren Y.Y. (2018). Development of a rotating electric field conductance sensor for measurement of water holdup in vertical oil–gas–water flows. Meas. Sci. Technol..

[33] Maxwell J.C. (1882). A Treatise on Electricity and Magnetism.

[34] Moody L.F. (1944). Friction factors for pipe flow. J. Appl. Mech. Trans. ASME.

[35] Harmathy T.Z. (2010). Velocity of large drops and bubbles in media of infinite or restricted extent. AIChE J..

[36] Wallis G.B. (1970). Minimum critical velocity for one-phase flow of liquids, Industrial and Engineering Chemistry Process Design and Development. Ind. Eng. Chem. Res..

[37] Zuber N., Hench J. (1962). Steady State and Transient Void Fraction of Bubbling Systems and Their Operating Limit.

[38] Jin N.D., Zheng G.B., Chen W.P. (2007). Chaotic recurrence characteristics analysis of conductance fluctuating signal of gas/liquid two-phase flow. J. Chem. Ind. Eng..

